# A detached leaf assay for testing transient gene expression and gene editing in cowpea (*Vigna unguiculata* [L.] Walp.)

**DOI:** 10.1186/s13007-020-00630-4

**Published:** 2020-06-15

**Authors:** Martina Juranić, Dilrukshi S. K. Nagahatenna, Rigel Salinas-Gamboa, Melanie L. Hand, Nidia Sánchez-León, Weng Herng Leong, Tracy How, Natalia Bazanova, Andrew Spriggs, Jean-Philippe Vielle-Calzada, Anna M. G. Koltunow

**Affiliations:** 1 Commonwealth Scientific and Industrial Research Organisation (CSIRO) Agriculture and Food, Urrbrae, SA 5064 Australia; 2 Grupo de Desarrollo Reproductivo y Apomixis, UGA Laboratorio Nacional de Genómica para la Biodiversidad, CINVESTAV Irapuato, Guanajuato, Mexico; 3 Commonwealth Scientific and Industrial Research Organisation (CSIRO) Agriculture and Food, Black Mountain Laboratories, Canberra, ACT 2601 Australia; 4 Present Address: Centre for Crop Science, Queensland Alliance for Agriculture and Food Innovation (QAAFI), The University of Queensland, Brisbane, QLD 4072 Australia

**Keywords:** Genome editing, CRISPR/Cas9, Cowpea, Meiosis, Leaf, Transient assay, Plant reproduction

## Abstract

**Background:**

The legume cowpea (*Vigna unguiculata* L.) is extensively grown in sub-Saharan Africa. Cowpea, like many legumes has proved recalcitrant to plant transformation. A rapid transient leaf assay was developed for testing gene expression and editing constructs prior to stable cowpea transformation, to accelerate cowpea and legume crop improvement.

**Results:**

Attempts to develop a transient protoplast system for cowpea were unsuccessful. Leaflets from plants 3–4 weeks post-germination were age selected to establish a rapid *Agrobacterium* (Agro) infiltration-mediated transient system for efficacy testing of gene expression and CRISPR/Cas9 gene editing constructs. In planta, Agro-infiltration of leaflets with fluorescent expression constructs, resulted in necrosis. By contrast, Agro-infiltration of detached leaflets with an *Arabidopsis* (At) *ubiquitin3* promoter:*ZsGreen* construct, followed by culture on solid nutrient medium resulted in fluorescence in over 48% of leaf cells. Expression efficiency was leaf age-dependent. Three cowpea meiosis genes were identified for CRISPR/Cas9 gene-editing, with the forward aim of meiosis-knock out for asexual seed induction in cowpea. Constructs were designed and tested containing candidate gene-specific guide RNAs, expressed using either the cowpea or *Arabidopsis U6* promoters with *Cas9* expression directed by either the *Arabidopsis* 40S ribosomal protein or parsley *ubiquitin4*-*2* promoters. Leaflets were infiltrated with test gene-editing constructs and analytical methods developed to identify gene-specific mutations. A construct that produced mutations predicted to induce functional knockout of in the *VuSPO11*-*1* meiosis gene was tested for efficacy in primary transgenic cowpea plants using a previously established stable transformation protocol. *Vuspo11*-*1* mutants were identified, that cytologically phenocopied *spo11*-*1* mutants previously characterized in *Arabidopsis,* and rice. Importantly, a biallelic male and female sterile mutant was identified in primary transgenics, exhibiting the expected defects in 100% of examined male and female meiocytes.

**Conclusion:**

The transient, detached cowpea leaf assay, and supporting analytical methods developed, provide a rapid and reproducible means for testing gene expression constructs, and constructs for inducing mutagenesis in genes involved in both vegetative and reproductive developmental programs. The method and tested editing constructs and components have potential application for a range of crop legumes.

## Background

Cowpea (*Vigna unguiculata* [L.] Walp.), a grain legume crop which originated in Africa, improves soil fertility due to its ability to fix atmospheric nitrogen. It is a subsistence crop providing high-quality dietary proteins, and calories for over 200 million people [2]. Increasing yield and quality of cowpea seed remains a high priority in breeding programs which are focused on increased drought tolerance and early grain maturity [7, 15, 24]. Most cowpea genotypes are highly susceptible to *Maruca vitrata*, a pod borer which devastates crop yields and naturally resistant germplasm has not been identified. *Maruca*-resistant cowpea varieties containing the *Bacillus thuringiensis* (BT) gene have now been developed for Nigerian agricultural production, providing a transgenic platform for cowpea crop improvement [1].

The generation of *Maruca*-resistant cowpea varieties utilized a labor intensive, low efficiency transformation protocol with a regeneration frequency of less than 1% [35]. Cowpea, like other legumes is relatively recalcitrant to transformation. Cowpea transformation efficiencies have recently increased [8]. The newly developed genome and transcriptome resources, in combination with CRISPR/Cas9 gene editing can support progression of varietal improvement programs [17, 26, 40]. Gene editing together with the low efficiency transformation method has successfully uncovered roles of two cowpea centromeric histone H3 genes [19]. Cowpea has a generation time of up to 14 weeks or more, depending on the variety, there is limited information concerning the efficiency of gene editing vectors for cowpea, and designed target guides need testing for efficiency. A transient assay to rapidly pre-test gene expression and gene editing constructs, could accelerate the improvement of cowpea and related legume crops which can also exhibit long generation times.

Transient transformation assay systems using protoplast and/or *Agrobacterium* (Agro)-infiltration represent an efficient alternative to rapidly test gene expression in species with long life cycles or being recalcitrant to stable transformation. The strategy has been utilized with varying success in several legume species, including, *Phaseolus vulgaris* [31, 44], *Lotus japonicus*, soybean [44] and *Medicago trunculata* [34]. To date, there have been no reports of testing CRISPR/Cas9 constructs in legume transient assays. CRISPR/Cas9 mediated-gene editing was successfully deployed by hairy root transformation of cowpea [21], soybean [6, 43] and *Medicago trunculata* [29]. The key advantage of transient assays over hairy root transformation is that experimental results can be rapidly generated.

Our long-term goal is to alter cowpea reproduction from a sexual to an asexual mode to enable smallholder African farmers to economically save high-yielding hybrid cowpea seed for planting [9]. One step towards synthesis of asexual hybrids, requires engineering meiosis so that it resembles mitosis during male and female gamete formation. This phenotype, termed Mitosis instead of Meiosis (MiMe), can be achieved in *Arabidopsis* by the combined mutagenesis of specific meiosis genes, for example, *SPO11*-*1*, *REC8*, and *OSD1*. Mutation of *SPO11*-*1* impairs homologous recombination, mutation of *REC8* disrupts monopolar orientation of sister chromatids, and mutations in *OSD1* prevent the occurrence of a second meiotic division [11]. Mutagenesis of these and additional genes also induces a MiMe phenotype in rice [23, 28, 30, 47].

Here, we investigated the development of a cowpea transient system using protoplasts and also Agro-infiltration of leaves of different developmental stages in young plants. Agro-infiltration proved effective in detached leaflets of particular ages followed by culture on solid medium prior to analysis proved efficient for expression of a fluorescent constitutive reporter. Cowpea homologs of *SPO11*-*1*, *REC8*, and *OSD1* meiosis were identified and the detached leaf transient system was used to test various CRISPR/Cas9 gene-editing constructs for inducing mutations in these genes in leaf cell DNA. The transient system proved amenable for rapid testing of a number of vectors containing different guide RNAs where guides and *Cas9* were expressed using different promoters. Finally, the utility of the system for pre-testing of gene editing constructs was demonstrated with the regeneration of transgenic cowpea plants containing mutations in the *SPO11*-*1* gene and meiotic impairment leading to sterility.

## Results

### Cowpea transient expression system development

**Fig. 1:**
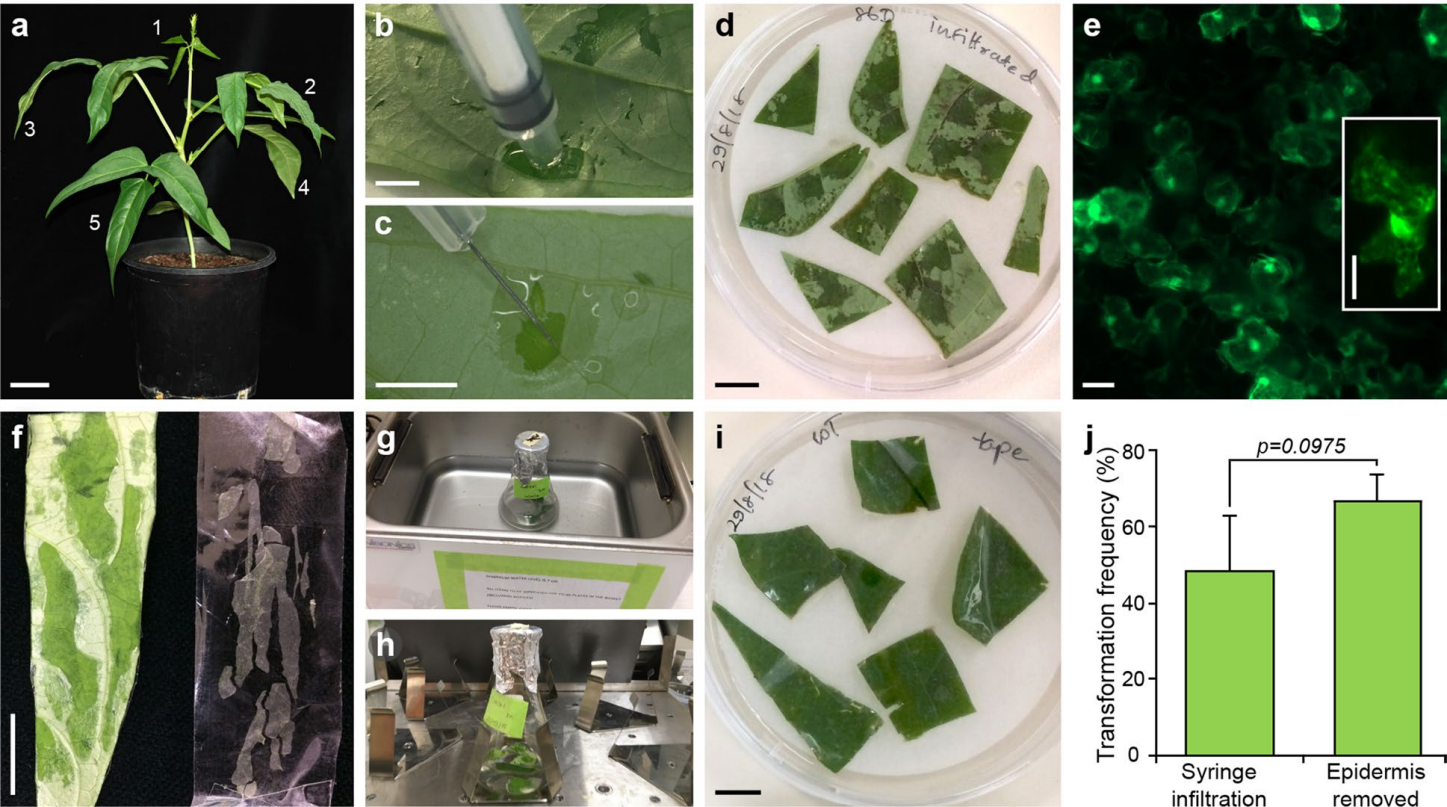
Development of the cowpea leaf transient expression system using a fluorescent reporter (*At*-*UBQ3*_*pro*_*:ZsGreen*). **a** A 3–4-week old cowpea plant showing the first five trifoliate leaves numbered from apex to base. The central terminal leaflets from trifoliate leaves at positions 3 and 4 were used in experiments. Direct injection of *Agrobacterium* suspension into cowpea leaves using a syringe **b** without or **c** with a needle. **d** Detached, leaflet pieces, infiltrated with *Agrobacterium* containing the constitutive *AtUBQ3*_*pro*_*:ZsGreen* reporter, resting on filter paper on top of solid “Medium 4”, infiltrated side in contact with filter paper. **e***AtUBQ3*_*pro*_*:ZsGreen* reporter fluorescence detected in infiltrated leaf cells 2 days after incubation. Inset shows an individual fluorescing leaf cell. **f** Removal of the lower leaf epidermis using cello tape. **g** Sonication of leaf pieces with epidermis removed in a flask containing *Agrobacterium* in a water bath at room temperature, followed by **h** shaking at 100 rpm for 30 min and, **i** incubation on filter paper on top of solid “Medium 4” (lower leaf side in contact with the filter paper). **j** Higher numerical frequencies of *AtUBQ3*_*pro*_*:ZsGreen* leaf cell fluorescence were obtained when the epidermis was removed, but this was not statistically significant. Transient transformation frequency was calculated as % of cells with fluorescence in the field of total cells (minimum of 200 counted cells) ± SD. Scale bars: **a** = 5 cm; **b**, **c** = 0.5 cm; **d**, **f** and **i** = 1 cm; **e** = 20 µm

Initially we tried to establish a transient protoplast system for cowpea. Two methods were trialed for protoplast release. One involved cutting cowpea leaves into strips [51] and in the other, the lower epidermis was peeled away using tape to expose mesophyll cells [49] prior to enzymic digestion to remove cell walls. Central terminal leaflets from cowpea trifoliate leaves at positions 1 to 5 on 3–4 week old cowpea plants, were used in these experiments (Fig. 1a). The efficiency of mesophyll protoplast release was age-dependent. Well-expanded terminal leaflets at leaf positions 3 and 4 produced up to 0.96 ± 0.42 × 10^5^ mesophyll protoplasts per gram of fresh tissue weight using the Wu et al. [49] method. Few protoplasts were released from the other tested leaflets. Protoplast viability was dependent on mannitol concentration with 0.6 M mannitol resulting in highest viability (50.3 ± 16.7%; Additional file 1: Fig. S1a–c). These protoplasts remained viable for 48 h, however, we failed to introduce a constitutive *AtUBQ3*_*pro*_*:ZsGreen* fluorescent reporter construct into mesophyll protoplasts via the polyethylene glycol-mediated transfection procedures described by Yoo et al. [51].

Leaf *Agrobacterium* (Agro) infiltration methods were then tested. *Agrobacterium* (AGL-1) cells containing a *AtUBQ3*_*pro*_*:ZsGreen* fluorescent reporter were resuspended either in liquid medium used for cowpea transformation or in tobacco infiltration medium, then directly infiltrated onto terminal leaflets at positions 3 or 4 on 3–4 week old plants using a syringe (“Methods”; [37]). Agro-infiltration in planta resulted in rapid leaf photo-bleaching, browning and senescence in sectors where the inoculum was applied irrespective of the *Agrobacterium* resuspension medium (Additional file 1: Fig. S1d). Expression of the *AtUBQ3*_*pro*_*:ZsGreen* reporter gene was successfully observed when detached cowpea terminal leaflets from positions 3 and 4 were Agro-infiltrated using transformation solution [35], blotted and then cultured for 2 days infiltration side down on filter paper on solid medium containing sucrose and nutrient media used in plant transformation (“Methods”; Fig. 1b–e).

A second, modified procedure was tested to examine if the transformation efficiency could be improved as measured by an increased number of fluorescent leaf cells/area. The lower epidermis was removed from central terminal leaflets sampled from leaves at positions 3 and 4, followed by sonication, shaking in an *Agrobacterium* solution and then subculture on solid medium (Fig. 1f–i; “Methods”). Comparison of transient efficiencies observed using both procedures showed that fluorescent cell frequencies ranged from 48 to 67% in a field of 200 cells. Statistical comparisons showed that there was no significant difference in transient transformation efficiency between both methods (Fig. 1j).

The simplest method of Agro-infiltration of detached leaves followed by culture on solid medium was, therefore, used in subsequent experiments to explore the utility of the system for pre-testing CRISPR/Cas9 gene editing constructs to induce mutations in specific cowpea meiosis genes.

### Identification of cowpea *SPO11*-*1*, *REC8* and *OSD1* meiosis genes

Cowpea homologs of the *Arabidopsis SPO11*-*1*, *REC8* and *OSD1* meiosis genes evident in genomes of cowpea IT86D-1010 [40] and IT97K-499-35 [26] were isolated and characterized with the forward aim of inducing mutations in them to establish a MiMe phenotype in transgenic cowpea (Additional file 6: Table S1). Each of the three genes are present in single copy in both accessions. *OSD1* and *REC8* showed high sequence conservation. *SPO11*-*1* was the most polymorphic with 57 single-nucleotide polymorphisms (SNPs) in exons and introns, and 7 indels in introns identified between the two accessions (Additional file 6: Table S1).

Comparisons of these three cowpea meiosis genes with homologs identified in seven other legumes indicated they are present in single copy in two other *Vigna* species: adzuki bean and mung bean (*V. angularis* and *V. radiata*, respectively; Additional file 1: Fig. S1 and Additional file 6: Table S2). Multiple copies of all three genes were found in diploid *Medicago trunculata* and *Lotus japonicus*. Three copies of *REC8* were evident in diploid common bean (*Phaseolus vulgaris*), two with 3′ end truncations. Two copies of *OSD1* and *REC8* genes were evident in soybean (*Glycine max*), which underwent whole genome duplication approximately 13 Myr ago [39]. Synteny analyses revealed that in legumes with single copies of *SPO11*-*1*, *REC8* and *OSD1*, each gene resides in a conserved locus with substantial collinearity and preservation of neighboring genes. In legumes with multiple gene copies, additional genes reside in regions lacking synteny, except for soybean which underwent whole genome duplication (Additional file 2: Fig. S2, Additional file 6: Table S3). Thus, these meiosis gene duplications are the likely result of local chromosome rearrangements (Additional file 2: Fig. S2c, f, i). Despite the copy number differences between the examined legumes, *OSD1*, *REC8* and *SPO11*-*1* are highly conserved in sequence and gene structure (Additional file 2: Fig. S2b, e, h and Additional file 6: Tables S3, S4).

Splicing variants were observed when the expressed sequences of cowpea *SPO11*-*1*, *REC8* and *OSD1* genes were isolated from cowpea IT86D-1010 female reproductive tissue. Splice variants were verified by sequencing cDNAs from whole ovaries at different temporal stages of female gametophyte formation (pre-meiosis, meiosis and female gametophytes formation; Additional file 3: Fig. S3, Additional file 6: Table S5). Three alternative splicing variants were observed for *REC8*, 10 for *SPO11*-*1*, and no variants were detected for *OSD1* in ovary tissue. None of the alternative splicing transcripts observed correlated with a specific developmental stage (Additional file 6: Table S5). Our in silico analyses suggest likely alternative splicing for *SPO11*-*1* in soybean, mung bean and pigeon pea (Additional file 6: Table S6). Alternative splicing for *SPO11*-*1* has been previously reported in *Arabidopsis*, rice, *Brassica rapa*, *Carica papaya* and *Physcomitrella patens* [18, 41]. Full-length, correctly spliced transcripts for all three genes were only detected in pre-meiotic and meiotic ovaries of IT86D-1010. These data suggest that the regulation of transcript splicing may be important to bring together meiotic components in reproductive cell types.

### SPO11-1, REC8 and OSD1 mRNAs and proteins are not restricted to cowpea meiotic cell types

**Fig. 2:**
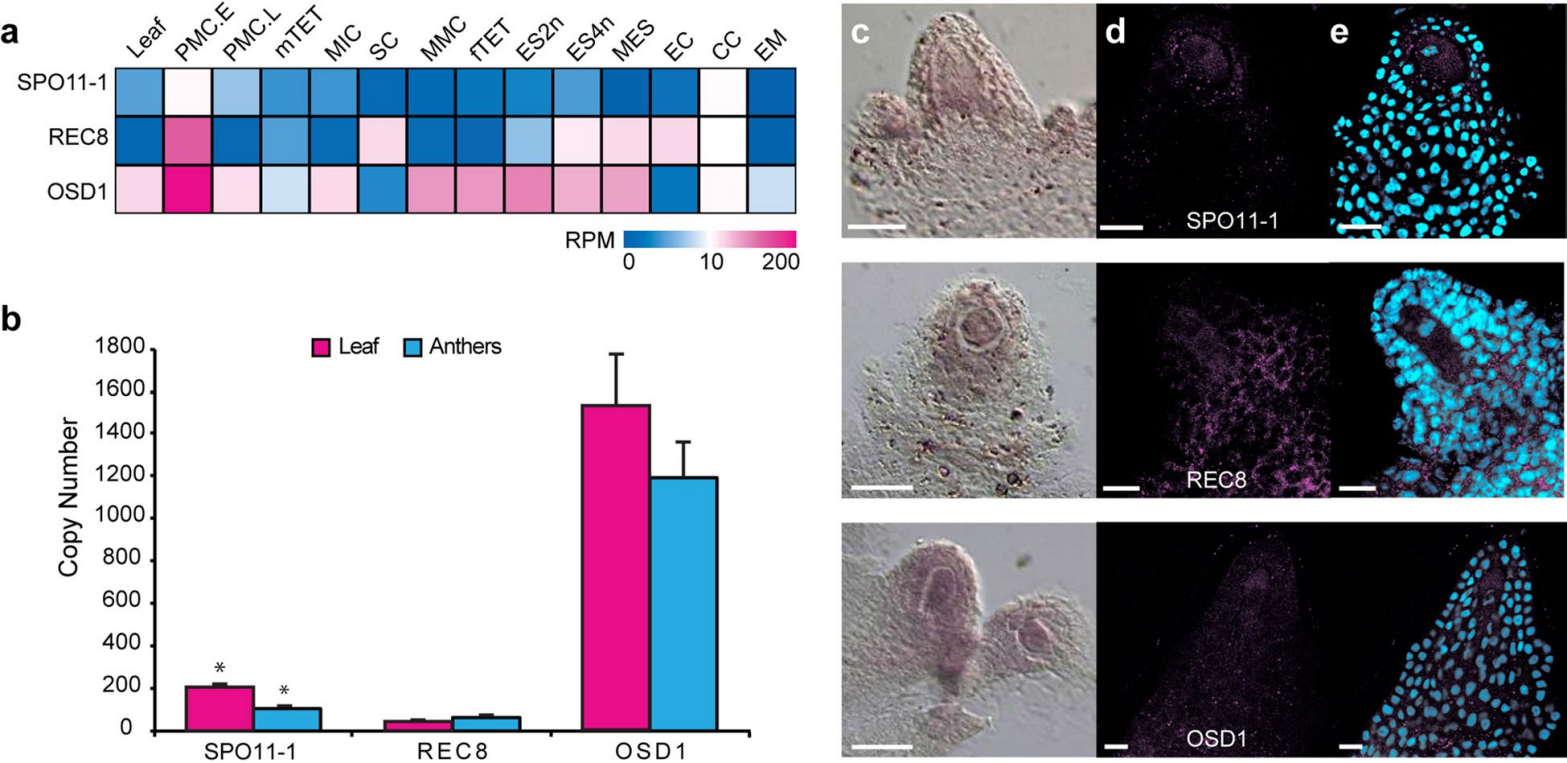
Expression of cowpea meiosis-related genes *SPO11*-*1*, *REC8* and *OSD1*, and protein localization in cowpea. **a** Expression of *SPO11*-*1*, *REC8* and *OSD1* genes in laser-captured cell types originating from cowpea reproductive tissues [17] represented as normalized read counts in a heat map. Pink indicates high abundance, and blue indicates low abundance. Additional file 6: Table S7 provides expression metrics. **b** Absolute expression levels of *SPO11*-*1*, *REC8* and *OSD1* in leaves and anthers collected at different developmental stages determined by quantitative PCR and normalized to the *ADF* gene. Asterisks indicate significant expression differences (*p < 0.05; Student’s t-test, X ± SE; n = 3 replicates). **c** Localization of meiosis gene transcripts (order as indicated in **d**), by in situ hybridization in developing ovules undergoing meiosis. Expression is evident in sporophytic and gametophytic tissues. **d** Protein localization determined by whole mount immunolocalization. **e** Detection of nuclei in images shown in **d** using propidium iodide (cyan). Scale bars: 10 µm. *ADF* actin depolymerizing factor 4, *PMC.E* early pollen mother cell, *PMC.L* late pollen mother cell, *mTET* male tetrads, *MIC* uninucleate microspore, *SC* mature pollen with sperm cells, *MMC* megaspore mother cell, *fTET* female tetrads, *ES2n* mitotic embryo sac with 2 nuclei, *ES4n* embryo sac with 4 nuclei, *MES* mature embryo sac at anthesis, containing the egg cell (EC) and central cell (CC), *EM* embryo

*SPO11*-*1*, *REC8* and *OSD1* transcripts were also detected during in silico analyses of cowpea male and female laser captured cell types undergoing meiosis. However, they were also detected in other cowpea laser captured reproductive cell types and in leaves, suggesting expression of these genes is not meiosis-specific (Fig. 2a; [17]). Analyses of the expression levels of the three genes by qRT-PCR in anthers and leaves, supported this observation with the detection of all three genes in leaves. *OSD1* showed highest expression levels in anthers and leaves (Fig. 2a, b, Additional file 6: Table S7).

The pattern of mRNA localization in cowpea ovules for the three genes was examined by in situ hybridization (Fig. 2c). All three genes showed mRNA localization in the megaspore mother cell (MMC) at the onset of meiosis, but also in other sporophytic cells of the developing ovule. *OSD1* mRNA was more restricted to the MMC and adjacent nucellar cells of the apical ovule primordia, including the L1 layer. The mRNA of *SPO11*-*1* and *REC8* were also localized in the developing inner integument. These results indicated that the expression of these genes is not restricted to female meiocytes in the ovule.

The pattern of protein localization of SPO11-1, REC8 and OSD1 was examined in ovules using polyclonal antibodies. To quantify protein expression, whole mount immunolocalizations were conducted for each protein and combined with 3D reconstruction to detect the overall fluorescent signal in images captured in the longitudinal plane of the developing ovule (n = 100 ovule primordia/protein). The number of cells present in the inner integument was used as a temporal reference for determining the developmental stage of the MMC [38]. SPO11-1, REC8 and OSD1 proteins showed a similar pattern of protein localization which was not restricted to meiotic cells, as proteins were detected in the MMC, companion nucellar, and occasionally integumentary cells (Fig. 2d, e, Additional file 4: Fig. S4).

To examine if the observed meiotic and non-meiotic cell type localization pattern of SPO11-1, REC8 and OSD1 proteins in ovules is specific to cowpea, REC8 expression was analyzed in the ovule primordium of *Arabidopsis thaliana*, using a specific antibody against the corresponding *Arabidopsis* REC8 protein. Interestingly, while 76% of the ovules analyzed showed AtREC8 localization restricted to the MMC, 22% showed expression in the MMC and adjacent nucellar cells, as was the case for most ovules of cowpea. The absence of signal in ovules of heterozygous *rec8/*+ individuals confirmed the specificity of the AtREC8 antibody (Additional file 4: Fig. S4a–c). These data suggest the existence of meiotic crosstalk occurring at the cellular intersection of the sporophytic and gametophytic generation in *Arabidopsis* and cowpea.

### Detection of CRISPR/Cas9-induced edits in cowpea meiosis genes using the transient leaf assay

Genome editing efficiency induced by the CRISPR/Cas9 system is influenced by several factors including target DNA site selection, single-guide RNA (sgRNA) design and Cas9 activity [25]. Different vector constructs were generated to target gene editing in the *SPO11*-*1*, *REC8* and *OSD1* genes using CRISPR/Cas9 with the aim of testing gene editing efficacy in the transient leaf assay prior to generation of constructs for stable cowpea transformation.

**Fig. 3:**
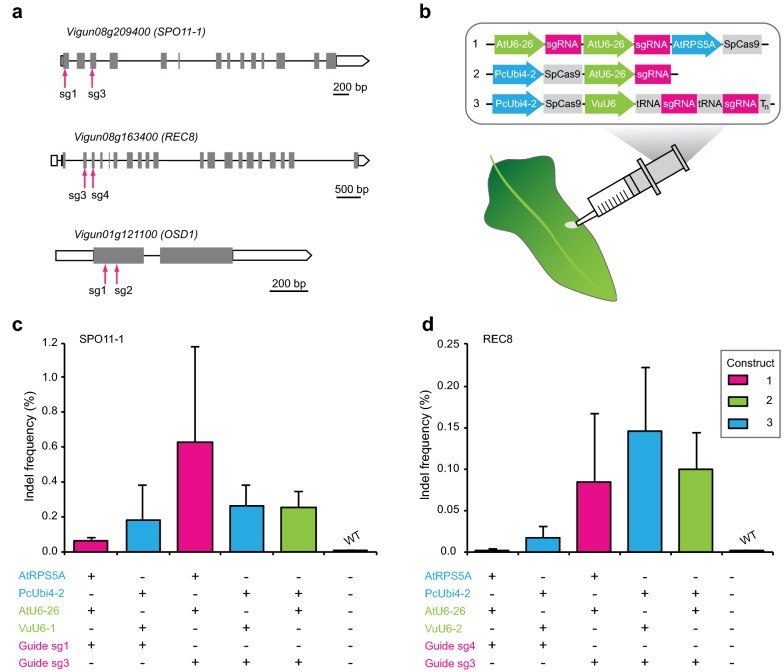
Analysis of genome-editing in cowpea leaves infiltrated with CRISPR/Cas9 constructs targeting *SPO11*-*1*, *REC8* and *OSD1.***a** Cartoons representing structures of *SPO11*-*1*, *REC8* and *OSD1* genes in a 5′-3′ orientation. The CRISPR/Cas9 target sites within each gene are indicated in magenta, exons are indicated by shaded rectangles, introns by intervening lines and untranslated sequences by unshaded rectangles. **b** Cartoon showing the three types of editing constructs (1–3) tested in the transient assay which differ in promoters driving *Cas9* and *sgRNA* expression (*AtRPS5A* vs *PcUbi4*-*2* and *AtU6*-*26* vs *VuU6*, respectively) and the number of guides targeting different genes (two or one). Construct 3 contains two sgRNAs that are expressed as a single polycistronic transcript, driven by *VuU6* promoter. T(7) is a 7nt terminator. **c** Mutagenic efficiency of guides targeting *SPO11*-*1* measured by indel frequency. Each bar represents the % mean value of at least seven independent transient assays ± SE. Non-significant differences from mean values between transiently transformed samples were found within the 95% confidence interval. Information below the X-axis indicates the components of the construct tested in the transient assay. The presence (+) or absence (−) of color-coded construct components match those in the diagram in **b**. **d** Mutagenic efficiency of guides targeting *REC8* measured by indel frequency. Guides targeting *OSD1* did not induce mutations. For further details refer to Additional file 6: Table S9

Two different sgRNAs were designed to typically target mutations the first exon in the 5′ region or the 5′ region of two consecutive exons in the 5′ regions each gene (Fig. 3a). The guides targeting mutations in *SPO11*-*1*, *REC8* and *OSD1* aimed to abolish expression of functional domains and often overlapped with corresponding DNA regions in *Arabidopsis* and rice homologs where mutations have resulted in functional knockouts [11, 16, 23, 47]. The on-target activity score for each guide was estimated using a predictive model ([12]; “Methods”). Vectors contained previously published CRISPR–Cas9 cassettes in which the *SpCas9* gene was driven either by the 40S ribosomal protein S5 (*RPS5A*) promoter from *Arabidopsis* [27] or the constitutive Ubiquitin4-2 promoter from *Petroselinum cripsum* (*PcUbi4*-*2*) [14]. The sgRNAs were expressed using either *AtU6*-*26* or *VuU6* promoters (Fig. 3b, “Methods”). The test constructs contained either one or two guide RNAs for each gene (Fig. 3b). Two of the 11 identified *U6* snRNA sequences (*VuU6*-*1* and *VuU6*-*2*) in the cowpea IT86D-1010 genome were also selected for testing in vectors used in the transient assay (Additional file 5: Fig. S5 and Additional file 6: Table S8). To further test the efficiency of multiplex gene editing, *SPO11*-*1* and *REC8* were targeted by a polycistronic tRNA-sgRNA construct consisting of two guides separated by tRNA motifs and driven by the *VuU6* promoter (Fig. 3b).

Genomic DNA was isolated from leaves infiltrated with the editing constructs after incubation on plates for 48 h. Infiltration with *Agrobacterium tumefaciens* bacteria without plasmid served as a wild type control. The regions flanking the sgRNA target sites in test and controls were sequenced using the Illumina platform. Assessment of the efficacy of mutation in the targeted regions using the transient assay was defined as the percentage of reads containing indels at the cleavage site. Observed mutations were typically single nucleotide insertions and deletions ranging from single to 18 nucleotides in length. Mutation frequencies of 0.04 to 0.5% for *REC8* and 0.05 to 3.9% were observed for *SPO11*-*1* in individual experiments (Additional file 6: Table S9). Taken together, the average editing efficiency of guides producing mutations in transient assays was around 1%, as calculated by taking the mutation efficiency at the target site from each independent sample and then averaging across all samples in experiments (Fig. 3c, d). By contrast, a comparative mutation frequency of not greater than 0.02% was detected in the control samples and also samples containing constructs targeting *OSD1* editing, all of which proved ineffective in inducing mutations.

By expressing two guides targeting a gene in the same vector, the editing efficiency of the individual guides could be comparatively examined in the assay to obtain a value for statistical significance. Mutagenic activities of *SPO11*-*1sg3* and *REC8sg3* were higher than the other pair of guides, but the differences were not statistically significant. Similarly, mutation efficiencies induced by guides expressed using the *VuU6*-*1* or *VuU6*-*2* promoters compared to the *AtU6*-*26* promoter were not statistically significant, nor the efficiency of the two promoters *PcUbi4*-*2* and *AtRPS5A* driving *Cas9* expression (Fig. 3c, d, Additional file 6: Table S9). A non-significant outcome with respect to editing efficiency in these tests was probably due to the observed variations in editing in different leaf samples and between experiments, which influenced statistical results (Additional file 6: Table S9). Nevertheless, all of the promoters utilized appeared functional in driving expression of linked genes in cowpea.

Importantly, the in silico methods used were relatively inefficient at predicting mutagenic efficiency of guides. For example, *SPO11*-*1sg3* induced targeted mutations at the highest frequency despite a low predicted activity score. *OSD1sg2* with the highest predicted activity score did not induce edits in the transient assay (Additional file 6: Table S9). These observations support the utility of the transient leaf assay for pre-testing constructs prior to proceeding with transgenic experiments.

### CRISPR/Cas9 induced *SPO11*-*1* gene editing disrupts meiosis in transgenic cowpea

The transient leaf assays indicated that a construct targeting gene editing in *SPO11*-*1* using two guides was potentially the most promising to test for *SPO11*-*1* knockouts in transgenic cowpea plants. In this construct, the *Arabidopsis RPS5a* promoter was linked to *Cas9* and two *AtU6*-*26* promoters enabled expression of the guide RNAs, sg1 and sg3. These guides facilitated deletions and insertions in *SPO11*-*1*, initiating 25 base pairs from the *SPO11*-*1* start codon in exon 1 and also in the third exon in transient assays (Fig. 3a, b, Additional file 6: Table S9).

The construct was introduced into cowpea using the method of Popelka et al. [35] and ten primary transformed (T0) plants were generated. Genomic DNA was extracted from the leaves. Targeted regions were then amplified and Illumina sequenced to assess the frequency and nature of the mutated sequences. Edits were detected in four of the ten T0 plants (Additional file 6: Table S10). In two of the four T0 lines, less than 0.5% of the genomic DNA was mutated (12A.1 and 12B.1; Additional file 6: Table S10). Mutations in both plants occurred at only one target site directed by the guide *SPO11*-*1sg3* in exon3. A higher mutation frequency was typically observed in the transient assay at the site directed by this guide (Additional file 6: Table S10).

**Fig. 4:**
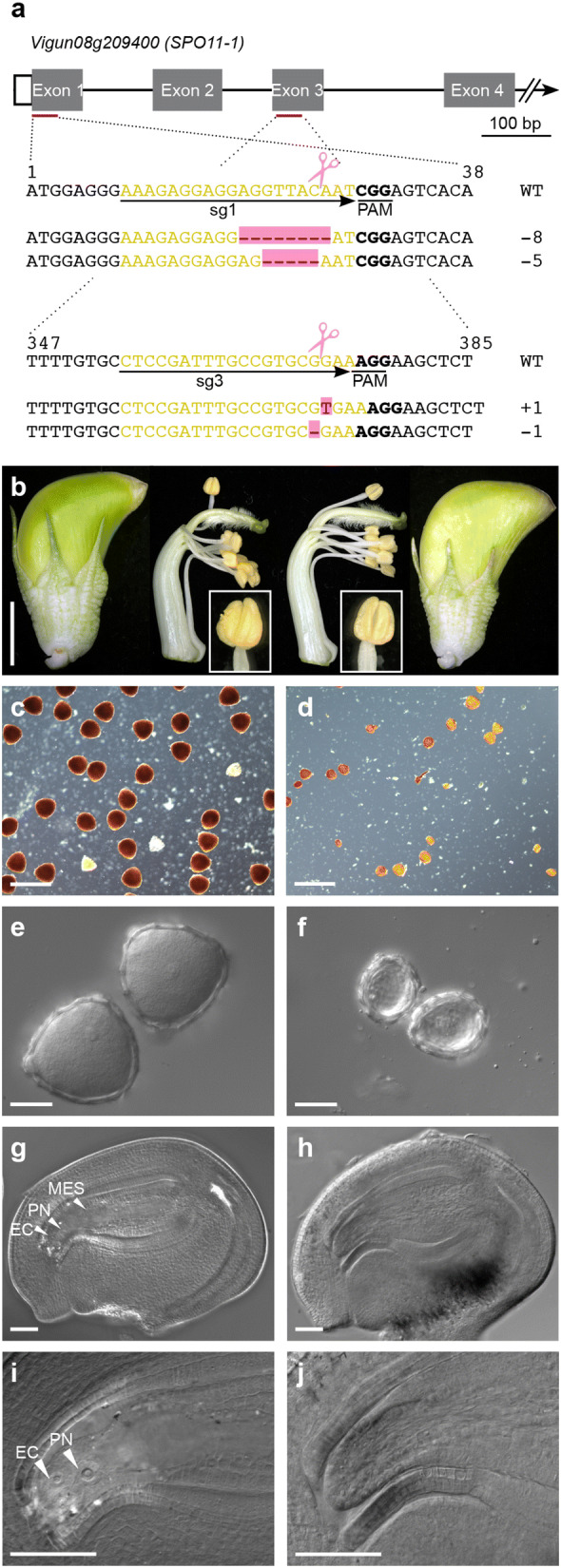
Male and female infertility phenotypes of the biallelic *Vuspo11*-*1* mutant. **a** Diagram of partial *SPO11*-*1* gene structure (four of 15 exons in grey) and the gene editing target sites in exon 1 and exon 3 in yellow. The protospacer adjacent motif (PAM) site is underlined and in bold. The Cas9 cleavage site is indicated with scissors and sequences altered in the biallelic *Vuspo11*-*1*-edited plant are shown in pink. **b** Floral buds of cowpea IT86D-1010 at stage 1F-IX [38], isolated gynoecia and anthers of wild-type at left and the biallelic *Vuspo11*-*1* mutant, right. Insets show anthers of WT (left) and *Vuspo11*-*1* mutant (right). **c** Viability of pollen grains at stage 1F-IX using iodine-potassium iodide staining WT and **d** biallelic *Vuspo11*-*1* mutant pollen grains. Intense dark brown indicates viable pollen. **e** WT and **f***Vuspo11*-*1* mutant pollen grains observed using DIC microscopy. **g**–**j** Mature female gametophytes showing normal development in WT **g** and **i**, and degeneration in *Vuspo11*-*1* mutant ovules **h** and **j**. Scale bars **b** = 0.5 cm, **c**, **d** = 100 µm, **e**, **f** = 20 µm, **g**, **h** = 50 µm. *CC* central cell, *EC* egg cell, *MES* mature embryo sac, *PN* polar nuclei

The other two primary transformants (1A.1 and 1A.2) were identified as mutants with biallelic edits in both exons 1 and 3 of the *SPO11*-*1* gene (Fig. 4a, Additional file 6: Table S10). This was determined by the presence of different mutations on both alleles at each target site with each occurring at 50% frequency (Additional file 6: Table S10). Sequence analyses of tissues isolated from the plants, at different time points confirmed these mutations were widespread throughout the plant body. These mutations (Fig. 4a) were predicted to cause translational frameshifts and introduction of stop codons in either exon 3 or exon 4. The predicted truncated *Vuspo11*-*1* mutant protein would be non-functional lacking the DNA binding domain (PF04406) encoded by exons 3–5. These plants appeared to be clones because they contained identical mutations and only one of these plants (1A.1; Additional file 6: Table S10) survived transfer to soil.

Developmental analysis of the T0 plant with biallelic edits in *SPO11*-*1* (Fig. 4a, b) indicated vegetative growth indistinguishable from untransformed cowpea plants. However, flowers abscised in comparison to untransformed controls and the plant continued to flower for an extended period. This is commonly observed in plants with induced reproductive sterility [42]. The other two transformants (12A.1 and 12B.1) with low mutation rate at only the exon 3 target site in the *SPO11*-*1* gene set pods with seed.

The mature anthers in the *Vuspo11*-*1* biallelic mutant were thin and shriveled. They dehisced small amounts of pollen which was variable in size and shape and nonviable following vital staining (Fig. 4c, d). Analyses of ovules in maturing flowers showed aborted female gametophytes suggesting male and female sterility was the cause of seed set failure in the transformant (Fig. 4g–j). The two T0 lines with low frequency of edits only in *SPO11*-*1* exon 3 exhibited wild type male and female gametophyte development.

**Fig. 5:**
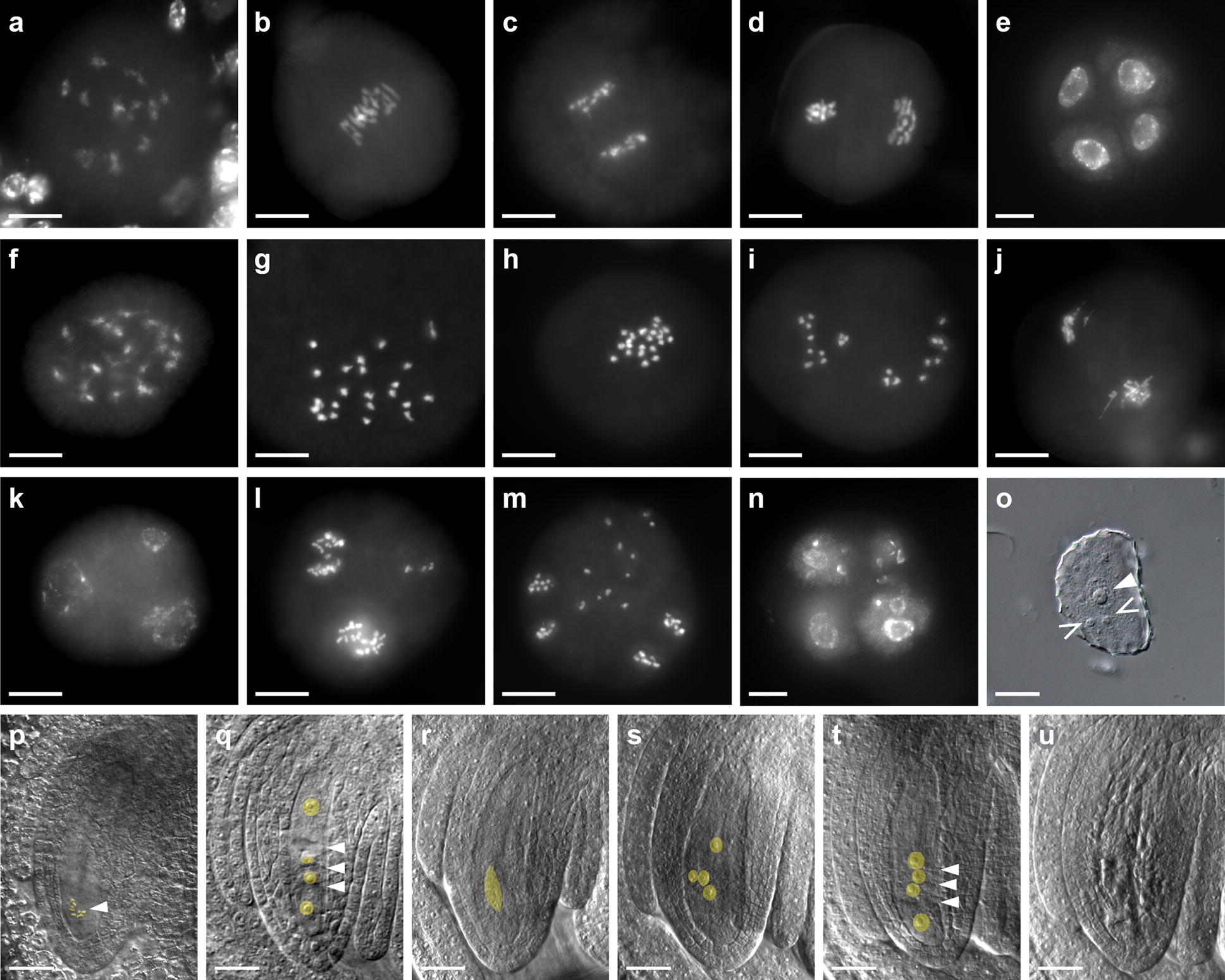
Analyses of male and female meiosis in the gene edited *Vuspo11*-*1* mutant relative to wildtype. **a**–**e** DAPI staining of wild-type male meiocytes (n = 22). **a** Diakinesis stage. **b** Metaphase I with 11 aligned bivalents. **c** Anaphase I. **d** Metaphase II with 11 sister chromatids separating to each pole. **e** Tetrads with decondensed chromatin. **f** to **n** DAPI staining of male meiocytes in the *Vuspo11*-*1*-edited plant. **f** Diakinesis stage, bivalent formation is not observed. **g** At the end of the prophase, 22 fully condensed chromosomes are observed. **h** Metaphase I with 22 univalents. **i** Random segregation of unpaired chromosomes at anaphase I. **j** Telophase I with micronuclei and chromosome loss. **k** Triads are observed at the end of meiosis I. **l**, **m** Second meiotic divisions result in sectors with variable number of chromosomes. **n** Tetrads with unbalanced chromosome numbers (depicted) and polyads with more than four nuclei are common (not shown). **o** Released microspore with one nucleus (arrowhead) and two micronuclei (open arrowhead; DIC microscopy). **p** DIC microscopy of female meiosis at metaphase I in wild-type with 11 bivalents indicated by the arrowhead. **q** Wild-type linear tetrad of female megaspores. Transverse cell walls separating megaspores are marked with arrowheads. **r**–**u** DIC microscopy of female meiosis in the *Vuspo11*-*1*-edited plant. **r** A meiotic spindle during anaphase I highlighted in yellow. **s** Cytokinesis does not occur at the end of meiosis in some of ovules. Nuclei are highlighted in yellow. **t** Mutant linear tetrad with four megaspores. Arrowheads are pointing towards incomplete cell plates. **u** Aborted megaspores. Scale bars: **a**–**n** = 10 µm, **o**–**u** = 20 µm

Knockout mutations in *SPO11*-*1* result in male and female sterility as homologous chromosome pairing and recombination are disrupted in meiosis [16, 42]. Meiosis was examined in the biallelic *Vuspo11*-*1* mutant and in untransformed plants to confirm if the observed sterility correlated with meiotic defects evident in *spo11*-*1* mutants characterized in other species (Fig. 5). In contrast to male gametogenesis in wild-type cowpea anthers where alignment of the eleven homologous chromosome bivalents was observed on the metaphase plate in metaphase I (Fig. 5b), 22 condensed univalents were observed in the biallelic mutant (Fig. 5f–h). As meiosis proceeded in the mutant, random segregation of univalents was observed (Fig. 5i–m) resulting in a variable number of chromosomes in the tetrads formed, and or, leading to the formation of more than four microspores (polyads) of different size and chromatin content, in addition to micro-nuclei (Fig. 5n). Examination of female meiosis in the *SPO11*-*1* gene edited mutant also showed alterations relative to the wild type, although it was more difficult to follow steps of meiotic progression (Fig. 5p–u). Functional megaspores typically aborted post-meiosis, resulting in sterile embryo sacs. This may be a consequence of aneuploidy arising from aberrant meiosis (Fig. 5u). Collectively, these data support the conclusion that reproductive sterility in the edited, phenotyped T0 plant is a consequence of defects in bivalent formation resulting from the functional knockout of the *SPO11*-*1* gene in cowpea.

## Discussion

Breeding programs targeting cowpea improvement are focused on increasing seed yield. Collectively this involves improvements in a number of physiological and developmental pathways including those promoting water use efficiency, stress and disease resilience, increased photosynthetic capacity and manipulating reproductive processes for flowering time and high seed set. Identification of critical genes, and use of synthetic biology approaches, involving targeted mutagenesis and relevant promoters to direct gene expression are key elements in combination with breeding programs supporting cowpea yield increases.

Meiosis needs to be altered to facilitate the induction and use of asexual reproduction in cowpea to preserve complex traits including hybrid vigor and accelerate breeding [9]. We therefore identified cowpea *SPO11*-*1*, *REC8*, and *OSD1* meiosis genes for collective gene editing to induce mitosis from meiosis, or the MiMe phenotype [11]. Analyses of the expression of these genes in cowpea and protein localizations, indicated that gene products are not specific to meiotic cells. This has been observed for meiosis associated genes in other species and suggests that cell-specific factors and protein–protein interactions are critical for enabling and regulating meiotic processes [10]. The sequence and phylogenetic analysis of these three cowpea meiosis genes with those identified in seven other legumes provided information on conserved regions. These data, in conjunction with that from mutant analyses in other species guided the choice of regions to target for gene editing.

Here, a simple transient assay utilizing Agro-infiltration in detached cowpea leaves was developed which enabled testing of gene expression in leaves and pre-testing of gene editing constructs for the forward induction of MiMe in transgenic cowpea. Unlike many other examples of in planta Agro-infiltration systems for transient gene expression, in planta Agro-infiltration of the cowpea leaves tested here, caused necrosis and wilting leaves. This may have related to the relatively young leaf stages used in combination with the bacterial inoculum concentrations. Necrosis was overcome by placing the detached, infiltrated and cut leaves on solid medium for 2 days. The use of the younger, softer and almost fully-expanded leaf tissue may have contributed to the high levels of fluorescence in leaf cells using the constitutive reporter gene. The same leaf stages were used in gene editing tests. It is unclear if leaf age also promoted editing efficiencies, as this was not examined. Nevertheless the ability to use young, fully-extended leaves 3–4 weeks after seed germination, increases the rate of result turn over.

Custom guide RNAs (sgRNAs) to target meiosis gene mutagenesis were designed using computational tools [12, 32]. However, the prediction accuracy of computational tools does not necessarily reflect experimental success [48]. This was also evident in this study as the predicted theoretical efficiencies of different guides resulted in different editing efficiencies using the transient system contrary to their predicted theoretical efficiency by computational tools.

The transient analyses collectively indicated that the guide sequence appears to be most important factor influencing the mutation efficiency of CRISPR/Cas9 system in the transient cowpea assay. A number of different promoters were examined in the transient cowpea assay that clearly enabled the expression of both *Cas9* and the guides. A significant effect of particular promoters enabling increases in mutagenic frequency was not evident from the set of transient analyses conducted here. Whether this proves to be the case in stable transgenics will be determined experimentally as greater numbers of gene edited transgenic cowpeas are generated.

The disruption of SPO11-1 function was induced by CRISPR/Cas9 gene editing in a regenerated T0 cowpea using a vector which displayed high mutation frequency in the transient leaf assay. Biallelic mutations in exon 1 and exon 3 of cowpea *SPO11*-*1* resulting in defects in meiosis leading to complete male and female sterility in the T0 plant. Cytological analyses during male and female meiosis indicated that meiotic defects phenocopied those which have been previously observed in *Arabidopsis*, rice and maize *spo11*-*1* mutants [36, 42, 52]. The induced meiosis defects included the presence of univalents (individual chromosomes) instead of bivalents (homologous chromosome pairs), their random partitioning during meiosis formation of polyads and unbalanced tetrads leading to aneuploidy and unviable gametophytes. The induction of a *spo11*-*1* mutant phenotype in transgenic cowpea confirms the utility of the transient assay for pre-testing and predicting constructs which are likely to induce successful genome edits in reproductive tissues of transgenic cowpea. Mutations were not efficiently induced in constructs made to target cowpea *OSD1* gene tested here, and the efficiency of *REC8* targeting for complete knock out in gametogenic cells needs to be improved. Additional constructs remain to be developed and tested for functional knockout of these two genes. The most straightforward means to create cowpea transgenics with a MiMe phenotype would be to induce a knockout of *SPO11*-*1*, *REC8* and *OSD1* by CRISPR/CAS9 as already reported for rice [23, 47]. Although recent reports suggest that new stable protocols based on in vitro cultivation of embryonic axis can result in a significant increase of cowpea transformation efficiency [8], CRISPR/CAS9-based editing requires time consuming efforts for selecting construct variants yielding the best functional results. The detached transient leaf assay developed here will facilitate selection of constructs for generation of cowpea transgenics exhibiting the mitotic MiMe phenotype.

## Conclusions

The detached leaf transient cowpea method is economical and simple in comparison to bombardment and protoplast transient methods. The method could potentially be applied to examine subcellular targeting to nucleus and/or cytoplasmic compartments. Conceivably, it may also be feasible to use antibodies to detect location of proteins formed from transiently expressed genes utilizing this method. However, this remains to be determined. The cowpea methods may also have application for testing gene expression, gene editing and protein localization in other legumes which are typically recalcitrant to transformation and have long generation times.

## Methods

### Plant materials and growth conditions

Cowpea (*Vigna unguiculata* L. Walp. cv. IT86D-1010) seeds were kindly provided by IITA (International Institute of Tropical Agriculture), Nigeria. Plants were grown under greenhouse conditions in the Australian Plant Phenomics Facility (APPF), Adelaide, Australia and Irapuato, Mexico as described previously [38].

### Cowpea leaves sampled for establishing transient protocols

Cowpeas form trifoliate leaves which have two asymmetrical side leaflets and one central, symmetrical terminal leaflet. Typically, five trifoliate leaves form after 3–4 weeks growth in aforementioned conditions. Four terminal leaflets were sampled for experiments from trifoliate leaves with position numbers beginning from the top of the plant (Fig. 1a). Leaves were sterilized in 70% ethanol for 2 min and rinsed two times with a tenfold volume of sterile distilled water and blotted with sterile paper, prior to further treatments below.

### Attempts at PEG-mediated transformation of mesophyll protoplasts generated from central terminal cowpea leaflets

Mesophyll protoplast isolation was conducted using sterilized central terminal leaflets from leaflet positions 1 to 5 (Fig. 1a) based on the protocol described by Yoo et al. [51] with slight modifications. Prepared leaf sections were quickly immersed in 5–10 ml enzyme solution (1.5% cellulase “ONOZUKA” R-10 (Yakult), 0.4% macerozyme R-10 (Yakult), 20 mM KCl, 20 mM MES, EDTA free protease inhibitor, 10 mM CaCl_2_, 5 mM DTT and 0.1% BSA, pH 5.7), upper epidermis side up with different concentrations of mannitol (0.4 M or 0.6 M). Leaf sections were vacuum infiltrated for 20–30 min in the dark and incubated with the cell wall hydrolysing enzymes for overnight at 25–30 °C with gentle shaking at 40 rpm. Filtering and subsequent centrifugation of the solution were conducted according to the instructions provided by Yoo et al. [51]. Protoplast density was calculated using a hemocytometer under a microscope and protoplasts were resuspended in MMG solution (4 mM MES, 0.4/0.6 M mannitol and 15 mM MgCl_2_, pH 5.7) to obtain the desired cell density (1–4 × 10^4^). The viability of protoplasts was detected by staining with 0.01% fluorescein diacetate (FDA) and incubating 5 min in the dark on ice. Protoplast viability (%) was determined as follows: protoplast viability (%) = (number of fluorescent protoplasts in view/total number of protoplasts in view) × 100%. Freshly prepared protoplasts were used for PEG transfection according to the instructions by Yoo et al. [51].

### *Agrobacterium* infiltration of cowpea leaves in planta

Relevant gene constructs transfected into *Agrobacterium* strain AGL1 were streaked out onto Luria broth (LB) agar supplemented with appropriate antibiotics and incubated for 2 days at 28 °C. A single colony from the plate was inoculated into 5 ml of liquid LB media with appropriate antibiotics and incubated for 24 h at 28 °C with shaking at 200 rpm. Two millilitre of culture was used to inoculate 20 ml of liquid LB media with appropriate antibiotics and incubated for additional 24 h at 28 °C and 200 rpm. *Agrobacterium* cells were pelleted for 5 min at 3900 rpm.

For Agro-infiltration in planta the pellet was resuspended in either: (i) a tobacco infiltration buffer containing 10 mM MgCl_2_, 10 mM MES, pH 5.6 to obtain an A_600_ of 0.6, and acetosyringone was added to 100 μM [37] or (ii) “Medium 4” used in a stable cowpea transformation [35]. The inoculum was incubated for 2–3 h at 28 °C and a 1 ml insulin syringe, without needle, was filled and used to infiltrate central terminal cowpea leaflets in planta. Typically leaflets from positions 3 and 4 from the top of a 3–4-week-old plant were infiltrated (Fig. 1a) by gently pressing the 1 ml syringe against the lower epidermis exerting counter-pressure with fingers on the other side of the leaf. Infiltration was observed as a spreading “wetting” area on the leaf.

### Transient transformation of detached cowpea leaves by in vitro *Agrobacterium* infiltration and culture on solid media

*Agrobacterium* cultures for transient transformation of detached leaves were prepared as for in planta infiltration above, except pellets were resuspended in liquid “Medium 4” [35] to obtain an A_600_ of 0.6. The inoculum was then incubated for 1 h at 28 °C with shaking at 200 rpm. Central terminal cowpea leaflets from positions 3 and 4 (Fig. 1a) were detached from the cowpea plant and sterilized as above. The lower epidermal sides of sterilized cowpea leaves were infiltrated using an insulin syringe with and without needle, until visible wetting was observed. Approximately 10–15 spots were infiltrated in the central part of each leaf (Fig. 1b, c). The leaf was blotted with sterile towels and cut into approximately 1–2 cm^2^ pieces and transferred to plates containing moist filter paper on solidified “Medium 4” containing 0.8% agar. The lower infiltrated side of each leaf piece was placed in contact with the agar and incubated at 25 °C for 2 days under 16 h light/8 h dark cycles and then observed for fluorescence (Fig. 1d) or processed for DNA extraction to examine edits in target genes (Fig. 3).

In a second method tested, a number of modifications were made. The lower epidermis was peeled away from each sterile leaflet according to the protocol described by Wu et al. [49]. The upper epidermal surface of the leaf was fixed to a 1-inch wide heavy-duty tape, while the lower surface was fixed to a 1 cm wide cello tape. The smaller tape was carefully removed to pull away the lower epidermal surface cell layer (Fig. 1e). The leaf was then cut into 1–2 cm^2^ squares (or approximately 5 pieces) and placed in a conical flask containing 25 ml of “Medium 4” with *Agrobacterium* inoculum (0.6 OD_600_) as above. Flasks were sonicated at 240 V, 0.6 A for 5 s in a sonicating water bath (FXP10, Unisonics) and incubated on a shaker for 30 min at 28 °C at 100 rpm (Fig. 1f, g). The suspension was poured off and leaf sections were blotted with sterile paper towel. Leaf sections were placed on top of moist sterile filter papers on solid Medium 1 with the lower side of the epidermis in contact with the filter paper and plates were incubated at 25 °C for 2 days under 16 h light/8 h dark cycles (Fig. 1h).

### Examination of fluorescence in cowpea leaves

Typically, three biological replicates with 10 leaf sections per replicate were examined for each independent experiment for assessing fluorescent reporter gene analyses. Leaf sections were mounted lower side up onto slides and covered with coverslips. They were assessed for the presence or absence of GFP reporter using a Zeiss Axio Imager M1 microscope with the Zeiss filter set 38 and transmitted-light bright field. Digital images were captured with an AxioCam MRm camera and ZEN 2 software (Carl Zeiss).

### Isolation of *SPO11*-*1*, *REC8* and *OSD1* genes in legumes

A BLAST(p) search was used to identify orthologues of the *Arabidopsis SPO11*-*1, REC8 and OSD1* genes (At3g13170, At5g05490, At3g57860) in eight legume genomes (*Vigna unguiculata* v1.0; *Vigna angularis* Va3.0; *Vigna radiata* Vr1.0; *Phaseolus vulgaris* v2.0; *Glycine max* Wm82.a2; *Cajanus cajan* Cc1.0; *Medicago truncatula* Mt4.0; *Lotus japonicus* Lj3.0) available at the Legume information system—LIS; http://LegumeInfo.org). Genomic and transcriptomic data of cowpea (genotype IT97K-499-35 and IT86D-1010 [26, 40]) were additionally used for in silico gene identification and confirmation. This search was extended to include all members of the REC8/RAD21 cohesin, SPO11/DNA topoisomerase VI and OSD1/UVI4 families in selected legume species. The maximum likelihood phylogenetic trees were built in SeaView v4.7 (http://doua.prabi.fr/software/seaview) as previously described [22]. Microsyntenic relationships were investigated using the Genomic Context Viewer built into the LIS and by comparing five nearest genes upstream and downstream of the gene of interest. Microsyntenic regions were then manually drawn in Adobe illustrator. Predicted intron–exon boundary sites were extracted from NCBI GenBank annotation data and gene structure schematic diagrams drawn using Adobe illustrator. All sequences used for phylogenetic comparisons and their accession codes are listed in Additional file 6: Tables S2, S4, S6.

### RNA isolation, expression analyses and molecular characterization of cowpea *SPO11*-*1*, *REC8* and *OSD1* genes

Total RNA was extracted from cowpea IT86D-1010 young leaves, pooled developing anthers and individual samples of whole gynoecia containing differentiated megaspore mother cells (MMC), female meiotic tetrads (fTET), and mature embryo sacs (MES) using the RNeasy Plant Mini Kit (Qiagen) following the manufacturer’s instructions. The reproductive calendars developed for cowpea by Salinas-Gamboa et al. [38] were used to harvest these reproductive tissue types. cDNA synthesis primed with oligo(dT)20 was carried out from 1 µg of DNase-treated RNA using the SuperScript III First-Strand Synthesis SuperMix (Invitrogen). To detect alternative splicing variants, RT-PCR was performed using equal amounts of cDNA originating from whole gynoecia at three stages with the Phusion High-Fidelity DNA Polymerase (NEB). The products were cloned into the pCR-Blunt vector (Invitrogen) and Sanger sequenced. Splicing variants were determined using multiple sequence alignments in Geneious v11.1.4 and compared with predicted gene models from reference cowpea genome (*Vigna unguiculata* v1.0, NSF, UCR, USAID, DOE-JGI, http://phytozome.jgi.doe.gov/) (Additional file 3: Fig. S3 and Additional file 6: Tables S5, S6). qRT-PCR reactions were performed on cDNA samples from leaf and anther tissues on a Roche LightCycler 480 Instrument II as described in Böttcher et al. [4]. Actin depolymerizing factor (ADF) 4 (*Vigun07g085400*) gene was used as a reference gene. Quantification cycle values were calculated based on the second derivative method included in the LightCycler 480 software, release 1.5.1 (Roche). The gene-specific primer pairs are listed in Additional file 6: Table S11. Transcriptional signatures of cowpea *SPO11*-*1*, *REC8* and *OSD1* genes during cowpea reproductive development were analyzed using normalized read counts generated from the laser captured cell transcriptome datasets as described in Gursanscky et al. [17]. Reads were aligned against the *Vigna unguiculata* IT97K-499-35 genome [26] using Biokanga as described [40] and uniquely aligned reads were counted for each gene.

### In situ hybridization

In situ hybridization (ISH) was performed as described in Vielle-Calzada et al. [45], with minor modifications. The sense and anti-sense 11-digoxigenin-UTP-labelled probes were made by amplifying genomic fragments for *VuSPO11*, *VuREC8*, and *VuOSD1* genes using the primers in Additional file 6: Table S11. The amplified fragments were cloned in PCRII TOPO (Invitrogen) and linearized with restriction enzymes cutting in the polylinker (*Xho*I *and Bam*HI, respectively); 1 μg was used as a template for probe synthesis. Sections at 12–15 μm thickness processed for in situ were analysed with a Leica DMRB microscope under brightfield and Nomarski illumination.

### Whole-mount protein immunolocalization

Immunolocalization was performed as previously described [38]. For cowpea, polyclonal anti-rabbit primary antibodies (LifeProtein) raised against selected peptides of VuSPO11 (MEGKRRRLQSESQAQSVILC), VuREC8 (CVSSVKAGDSAHSFPRPASEH), and VuOSD1 (CLKRTPSAKKAEREKRVRTL) were used at a 1:100 dilution (approximately 7.4 µg/µl). For *Arabidopsis*, an antibody raised against amino acids 178 to 353 of the SYN1/REC8 protein was used at a 1:250 dilution [5]. The meiotic protein ASY1 consistently expressed in the MMC during prophase I [38] and the absence of the primary antibody were used as positive and negative controls, respectively. Serial sections were captured on a laser scanning confocal microscope (Leica DM5500B and Zeiss LSM 510 META), with multitrack configuration for detecting PI (excitation with laser at 568 nm, emission collected using BP: 575–615 nm) and Alexa 488 (excitation with Argon laser at 488, emission collected using BP: 500–550 nm).

### Identification of *U6* promoters in cowpea

A BLAST(n) search was used to identify orthologues of the *Arabidopsis U6*-*26* non-coding gene (At3g13855) in cowpea IT86D-1010 genome assembly [40] and resulted in 11 hits with e-value above 1e−10 (Additional file 6: Table S8). Sequences were aligned in Geneious v11.1.4 using global alignment with 65% similarity. Two conserved elements common to plant *U6* promoters: an upstream sequence element (USE) and a TATA-like box were confirmed by comparison with the Arabidopsis *U6*-*26* promoter sequence [43, 46].

### Plasmid construction

Gateway^®^-compatible binary vector pOREOSAgw was created by insertion of a Gateway recombinational cassette from pEarleygate100 [13] into *Cla*I and *Kpn*I sites of pOREOSA vector backbone, which was kindly provided by Thomas J. Higgins (CSIRO). To facilitate subcloning of a gateway cassette, restriction sites were incorporated into primers used for amplification and subsequent cloning. The entry clone containing *At*-*UBQ3*_*pro*_*:ZsGreen* fusion was kindly provided by Shai J. Lawit and Marc Albertsen (Corteva Agriscience, Johnston, Iowa) and recombined into pOREOSAgw. For genome editing, single-guide RNA (sgRNA) sequences targeting exons of *OSD1, REC8* and *SPO11*-*1* genes, respectively, were designed using the CRISPRdirect web server https://crispr.dbcls.jp/ [32]. To minimize potential off-target activity, the targeting sequence predicted to have no more than one target in the *Vigna angularis* genome were selected. On-target activity of sgRNAs was scored by CRISPR Site Finder in-built in Geneious v11.1.4 based on a predictive model proposed by Doench et al. [12]. Scores were between 0 and 1, with a higher score denoting higher expected activity. The double-stranded sgRNAs with compatible overhangs were cloned into *Bsa*I digested pEN-Comaira.1 and pEN-Comaira.2. Third entry clone pEN-RC9.3 (a gift from Luca Comai, UC Davis; Lynagh et al. [27]) was modified by replacing the nopaline synthase (nos) terminator with *Pisum sativum* pea3A terminator, which was amplified from pDe-CAS9 (ABRC #CD3-1928; Fauser et al. [14]) and inserted into *Bam*HI and *Xba*I sites. Three entry clones were recombined into pOREOSAgw using 3-fragment MultiSite Gateway cloning generating a vector pOREOSAgw CRISPR/Cas9 containing two guides per target gene. To compare the mutagenesis efficiency of one guide per target gene, a sgRNA with higher specificity predicted by CRISPRdirect was selected for Gateway cloning into binary vector pDe-CAS9. Polycistronic tRNA-sgRNA cassettes driven by cowpea *U6* promoters were designed according to Xie et al. [50], synthesized as a fusion fragment by GenScript and subsequently cloned into pDe-CAS9. A 425 bp of the 5′ upstream region of the *U6* snRNA start site was used as a promoter. All gateway cloning reactions were performed with LR Clonase II Enzyme mix (ThermoFisher Scientific). All plasmid vectors were verified by sequencing and final constructs electroporated into *Agrobacterium tumefaciens* strain AGL1 for use in cowpea transient and stable transformations. Details of the primer and sgRNA sequences used in this study are described in Additional file 6: Table S11.

### Illumina sequencing of amplicons with custom library preparation

Genomic DNA was extracted from cowpea leaves using the DNeasy Plant kit (Qiagen, USA) according to the manufacturer’s recommendations. A two-step PCR approach combined with the dual index primers (Illumina, Inc) was performed to produce barcoded amplicons for MiSeq and pooled into a single library as described in Jacobs et al. [20]. A region of approximately 250 bp flanking the CRISPR cleavage sites in the cowpea *SPO11*-*1*, *REC8* and *OSD1* genes was targeted for sequencing. Reactions were run on a LightCycler^®^ 480 Instrument II (Roche Life Science) with addition of 1× SYBR™ Green I dye (ThermoFisher Scientific) to quantify and normalize reactions. Indexed libraries were purified using AMPure XP beads (Beckman Coulter Genomics, Danvers, MA, USA) and submitted for 150PE sequencing on the Illumina MiSeq platform at the Australian Genome Research Facility (AGRF, Melbourne). The size and quantity of the amplicon pool/library were assessed on Agilent TapeStation with the D1000 ScreenTape and the sequencing data demultiplexed using the Illumina bcl2fastq v2.20.0.422 pipeline. Within each sample, average of ~ 25,000 reads per sample was obtained. The reads were then processed and mapped to the reference cowpea genome for IT86D-1010 [40] using the Biokanga short-read aligner (https://github.com/csiro-crop-informatics/biokanga), using default parameters except for reporting up to 5 equally-best alignments for each read. Number of reads mapped to each gene were counted and for each sample, all the sequences were analysed for the presence of indels at the target site. Analysis of CRISPR editing data was also complimented with CRISPR RGEN Tool Cas-Analyzer software [33]. The most abundant, unique reads are reported in Additional file 6: Tables S9 and S10. The primer sequences used in library preparation are in Additional file 6: Table S11.

### Generation of transgenic cowpea plants containing *SPO11* gene editing constructs

Stable transformation of cowpea was achieved by *Agrobacterium*-mediated delivery of the CRISPR/Cas9 constructs to explants derived from the cotyledonary nodes of imbibed cowpea seeds as previously described by Popelka et al. [35] with an efficiency of less than 1%. Typically, 4000 bisected cotyledonary explants were prepared per construct and inoculated with suspension of *Agrobacterium tumefaciens* strain AGL1 at 0.8 OD_600_. Transgenic shoots were regenerated under a sequential kanamycin/geneticin regime at 150 and 20 mg/l, respectively at 26 °C with 16 h light photoperiod. Shoots developing healthy roots were transferred into 90 mm small pots containing sterilized soil mix (Van Schaik’s Bio-Gro Pty Ltd, Australia). Subsequently, these were acclimatized in the growth room at 22 °C with 16 h light photoperiod and 50% humidity for up to 4 weeks after which they were transferred to the glasshouse in larger pots. Transgenic plants were produced approximately 9 months after the inoculation with *Agrobacterium*. PCR was performed using DNA extracted from leaves of regenerated plants to confirm the presence of the *Cas9*, *nptII* and gRNA genes with the primers listed in Additional file 6: Table S11. Non-transgenic plants (IT86D-1010) were used as a negative control. All ten primary transgenic plants were screened for *SPO11*-*1* mutations by Illumina MiSeq amplicon sequencing with results reported in Additional file 6: Table S10.

### Phenotypic analyses of transgenic cowpea plants with confirmed edits in the *SPO11*-*1* gene

Three T0 plants with identified edits in *SPO11*-*1* gene (bi-allelic edits and edits with frequency below 0.5%) were further phenotypically analysed. Reproductive tissue was harvested from developing cowpeas flowers staged as per Salinas-Gamboa et al. [38]. For light microscopy, floral buds containing ovules and anthers were manually dissected and fixed in FAA as previously described [38]. The pollen viability tests were made a few minutes after placing fresh pollen grains in iodine-potassium iodide solution (0.5 g iodine and 1 g potassium iodide dissolved in 100 ml distilled water). Pollen grains stained dark (brown color) were viable, while nonviable pollen grains were pale or unstained. Images were captured using the Zeiss Axioskop 2 microscope equipped with Nomarski optics, Spot Flex color camera and Spot 5.1 software (Diagnostic Instruments, Inc). Meiotic chromosome spreads were prepared according to Bolanos-Villegas et al. [3] with modified enzyme digestion buffer: 0.2% cellulase “ONOZUKA” R-10 (Yakult), 0.3% pectolyase (Sigma), 0.3% cytohelicase (Sigma) in 10 mM citrate buffer at pH 4.5. Samples were mounted in Vectashield^®^ mounting medium with DAPI (Vector Laboratories) and examined using the Axio Imager M2 microscope (Carl Zeiss) with the Zeiss filter set 49. Digital images of spreads were captured with an AxioCam 506 camera and ZEN 2.6 software (Carl Zeiss).

## Supplementary information

**Additional file 1: Fig. S1.** Preliminary evaluation of different methods during development of the cowpea transient assay. a Effect of mannitol concentration on the viability of protoplast after enzyme treatment. Each bar represents the % mean value of viable protoplast counted under microscope in 15–20 different areas ± SD. At least 300 cells were counted per sample. Asterisks indicate statistical significance determined by Student’s t-test (**p < 0.01). b Protoplasts isolated from young fully extended cowpea leaves. c Protoplast stained with fluorescein diacetate (FDA) to test viability. FDA accumulates inside the plasma membrane of viable protoplast exclusively, while dead cells appear red in color from chloroplast autofluorescence. d Agro-infiltration in planta at the leaf stages 3–4 resulted in cell death response. Scale bars: B–C = 100 µm, D = 2 cm.

**Additional file 2: Fig. S2.** Comparative analysis of *OSD1*, *REC8* and *SPO11-1* homologs in eight legume species and Arabidopsis. a, d, g Maximum likelihood phylogenetic trees of a OSD1, d REC8 and g SPO11 proteins in eight legumes species and Arabidopsis. Analysis was done using amino acid (AA) sequence of full-length proteins. The number after the decimal point for a designated gene represents the splicing variant used for phylogenetic analysis. The bar at the bottom of the tree is the branch length, representing AA residue substitutions per site. Members of *Fabaceae* family fall into the temperate galegoid clade (cool season legumes) highlighted in dark green and the phaseoloid clade (tropical season legumes) highlighted in light green. Whole genome duplication (WGD) is indicated by an orange star in a. b, e, h Schematic of exon-intron organization of b *OSD1*, e *REC8* and h *SPO11* genomic sequences between legume species and Arabidopsis (exons, orange and blue rectangles; intron, black lines). Coding sequence is shown in orange, while 5’ and 3’-untranslated regions in blue. Genes are drawn to scale. Sequences are listed according to their phylogenetic relationships. c, f, i Graphic display of shared microsynteny of c *OSD1*, f *REC8* and i *SPO11* homologs. To simplify the visualization of genes from different species belonging to the same family, they were labelled with numbers and appear in the same color. The gene of interest (*OSD1*, *REC8* and *SPO11-1*) is positioned in the center grey box and highlighted in red. Synteny covers five genes upstream and downstream of the gene of interest. Chromosomes where synteny or a gene of interest is found are listed on the left in front of the microsynteny block. An asterisk in front of a chromosome number indicates that the original locus shares synteny with other species, however, a gene has translocated to different region. For information on gene families, refer to Additional File 6: Table S3. At, *Arabidopsis thaliana*; C.cajan, *Cajanus cajan*; chr, chromosome; Glyma, *Glycine max*; Lj, *Lotus japonicus*; Medtr, *Medicago truncatula*; Phvul, *Phaseolus vulgaris*; Vang, *Vigna angularis*; Vrad, *Vigna radiata*; Viung, *Vigna unguiculata*.

**Additional file 3: Fig. S3.** Splice variants of the *OSD1*, *REC8* and *SPO11-1* genes identified in ovules at different developmental stages in cowpea. Refer to Additional File 6: Table S5 for more details. a-c Exons are shown as grey rectangles, while introns are represented by black lines. Different splice variants are shown underneath the exon-intron organization of a *OSD1*, b *REC8* and c *SPO11-1*. d Semi-quantitative RT-PCR analysis of *OSD1*, *REC8* and *SPO11-1* mRNA. 1 µl of each cDNA was used for the PCR reaction. In the case of *SPO11-1*, a smear is visible representing a mixture of splicing variants. e Summary of results indicating the number of clones analyzed for each of three genes and the corresponding number of splice forms identified in reproductive tissues. Legend: fTET, female meiotic tetrads; MES, mature embryo sacs; MMC, differentiated megaspore mother cells.

**Additional file 4: Fig. S4.** Controls for immunolocalization and in situ hybridization and experiments. a, b Positive control localizing the expression of REC8 in *Arabidopsis*. a Wild type background showing consistent expression of REC8 protein in nucellar and gametophytic cells. b *rec8*/+ background not showing expression of the REC8 protein. c Frequency of REC8 localization in *Arabidopsis* and cowpea MMCs. d, e The immunolocalization negative control, without primary antibody. f-h Negative controls for in situ hybridization with a sense probe of f *SPO11-1*, g *REC8*, and h *OSD1*. Scale bars: 10 µm.

**Additional file 5: Fig. S5.** Alignment of 11 cowpea *U6* and *Arabidopsis U6-26* sequences. Conserved elements common to plant *U6* promoters; upstream sequence element (USE), TATA-box and *U6* small nuclear RNA (snRNA) sequence regions are highlighted in cyan, magenta and green respectively.

**Additional file 6: Table S1.** Genetic variation between two cowpea accessions (IT97K-499-35 and IT86D-1010) using Spriggs et al. [40] and Lonardi et al. [26] genome resources for comparison. **Table S2.** List of genes identified in this study. **Table S3.** Color codes and descriptions for gene families in microsyntenic region spanning 5 genes upstream and downstream of gene of interest. **Table S4.** SPO11-1, REC8 and OSD1 protein pairwise identities (in percentages) of legume species relative to cowpea. **Table S5.** Splice variant analysis of *SPO11-1*, *REC8* and *OSD1* genes in IT86D-1010 genotype. **Table S6.** Gene structure of *SPO11-1*, *REC8* and *OSD1* genes in legumes and *Arabidopsis*. **Table S7.** Normalized read count of *SPO11-1*, *REC8* and *OSD1* genes in laser-captured cell-type datasets. **Table S8.** Cowpea *U6* small nucleolar RNAs non-coding genes identified in this study. **Table S9.** Edits detected in *SPO11-1*, *REC8* and *OSD1* genes via the CRISPR/Cas9 transient assay in detached cowpea leaves. **Table S10.** Edits detected in *SPO11-1*-targeted T0 transgenic lines. **Table S11.** List of primers.

## Data Availability

All data generated or analysed during this study are included in this published article and its additional information files.
