# Supplementary figures and images for: A detached leaf assay for testing transient gene expression and gene editing in cowpea (Vigna unguiculata [L.] Walp.)
Source: Plant Methods. 2020 Jun 15;16:88. doi: 10.1186/s13007-020-00630-4 (PMC7296760; doi:10.1186/s13007-020-00630-4)

## Slide 1
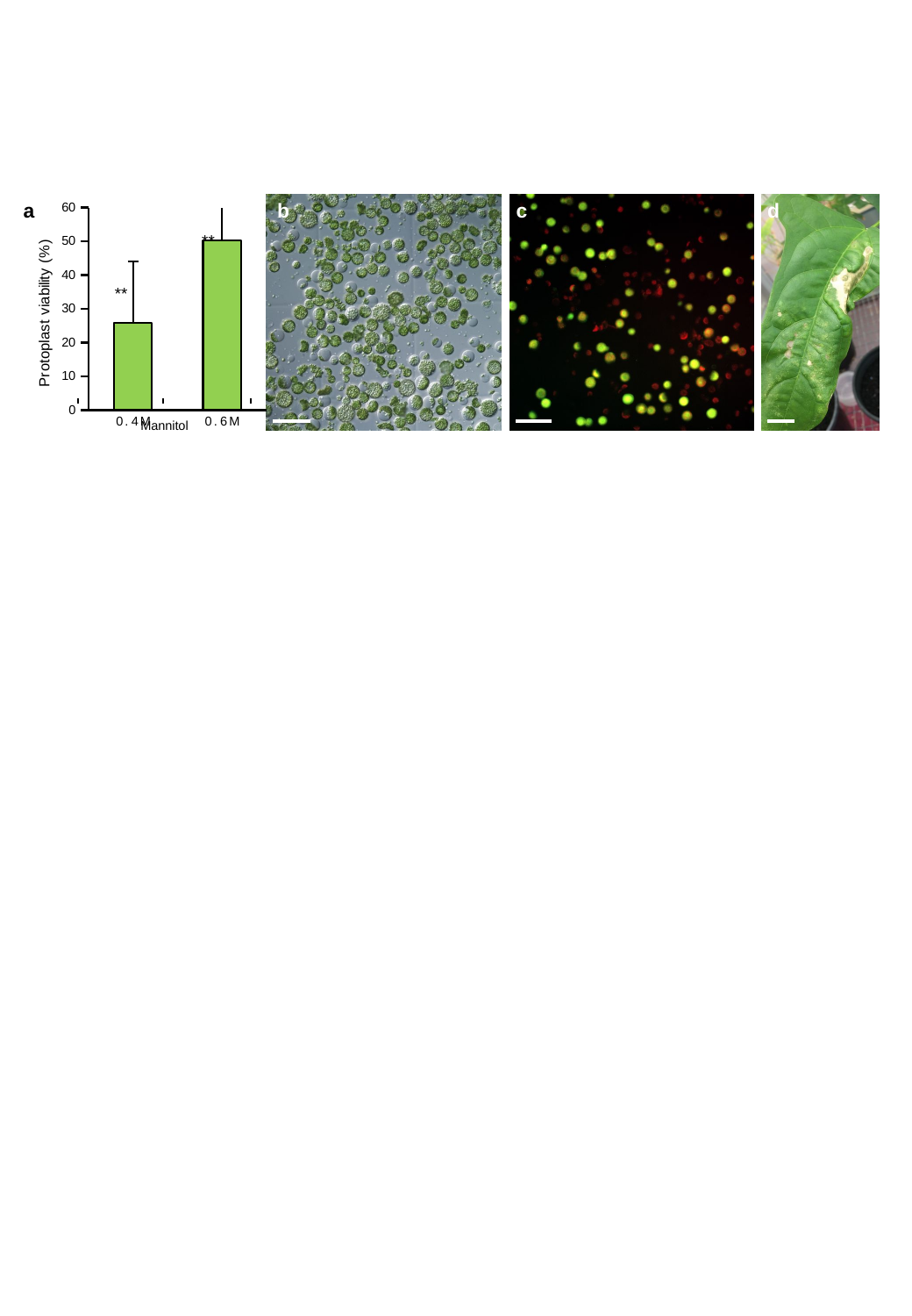

b
d
c
a
### Chart
| Category | Viability (%) |
|---|---|
| 0.4M | 25.809063281489752 |
| 0.6M | 50.31459553518377 |**
**
Mannitol

Supplement: Supplementary file 1 — Additional file 1: Fig. S1. Preliminary evaluation of different methods during development of the cowpea transient assay. a Effect of mannitol concentration on the viability of protoplast after enzyme treatment. Each bar represents the % mean value of viable protoplast counted under microscope in 15–20 different areas ± SD. At least 300 cells were counted per sample. Asterisks indicate statistical significance determined by Student’s t-test (**p < 0.01). b Protoplasts isolated from young fully extended cowpea leaves. c Protoplast stained with fluorescein diacetate (FDA) to test viability. FDA accumulates inside the plasma membrane of viable protoplast exclusively, while dead cells appear red in color from chloroplast autofluorescence. d Agro-infiltration in planta at the leaf stages 3–4 resulted in cell death response. Scale bars: B–C = 100 µm, D = 2 cm. [file 13007_2020_630_MOESM1_ESM.pptx]

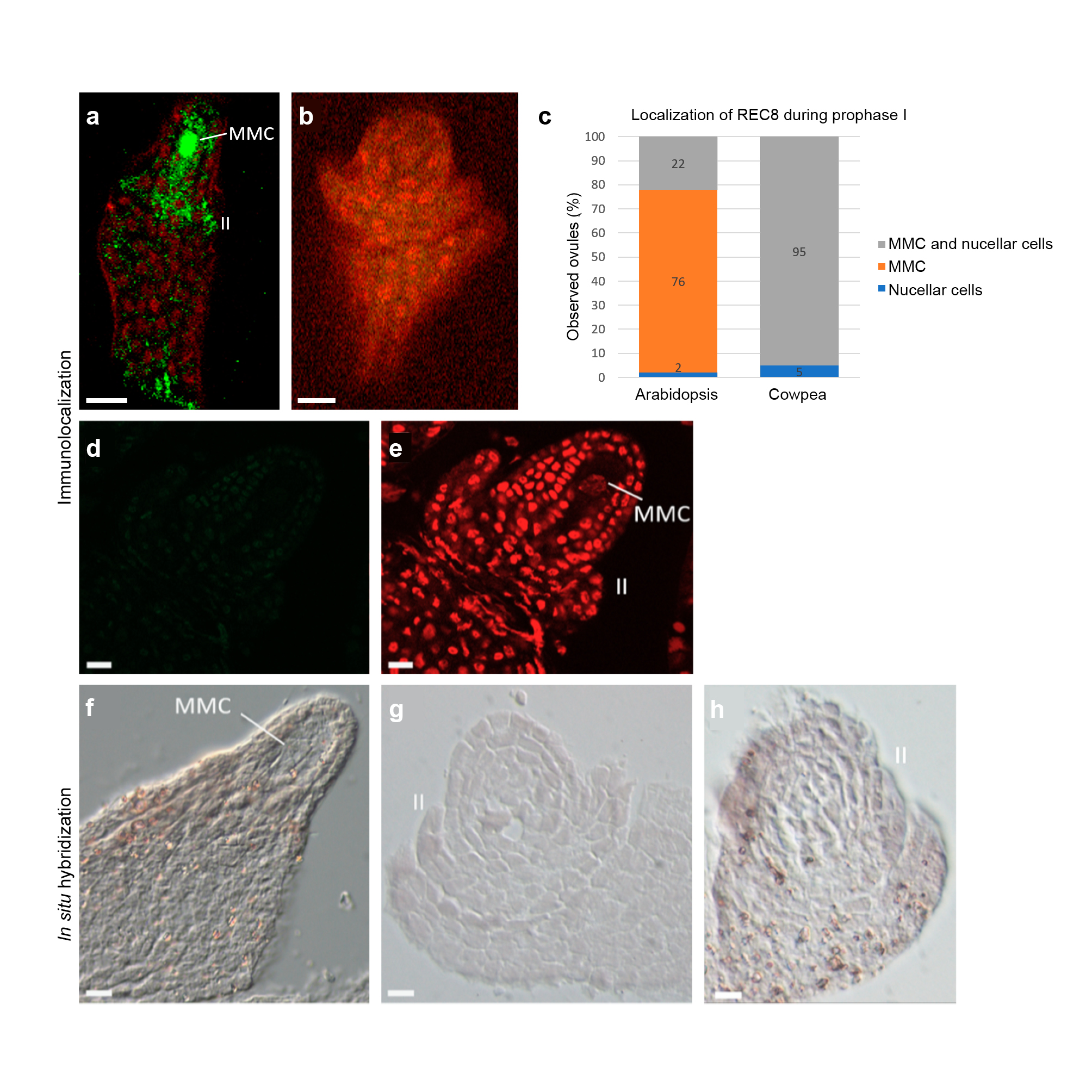

Supplement: Supplementary file 4 — Additional file 4: Fig. S4. Controls for immunolocalization and in situ hybridization and experiments. a, b Positive control localizing the expression of REC8 in Arabidopsis. a Wild type background showing consistent expression of REC8 protein in nucellar and gametophytic cells. b rec8/+ background not showing expression of the REC8 protein. c Frequency of REC8 localization in Arabidopsis and cowpea MMCs. d, e The immunolocalization negative control, without primary antibody. f-h Negative controls for in situ hybridization with a sense probe of f SPO11-1, g REC8, and h OSD1. Scale bars: 10 µm. [file 13007_2020_630_MOESM4_ESM.jpg]

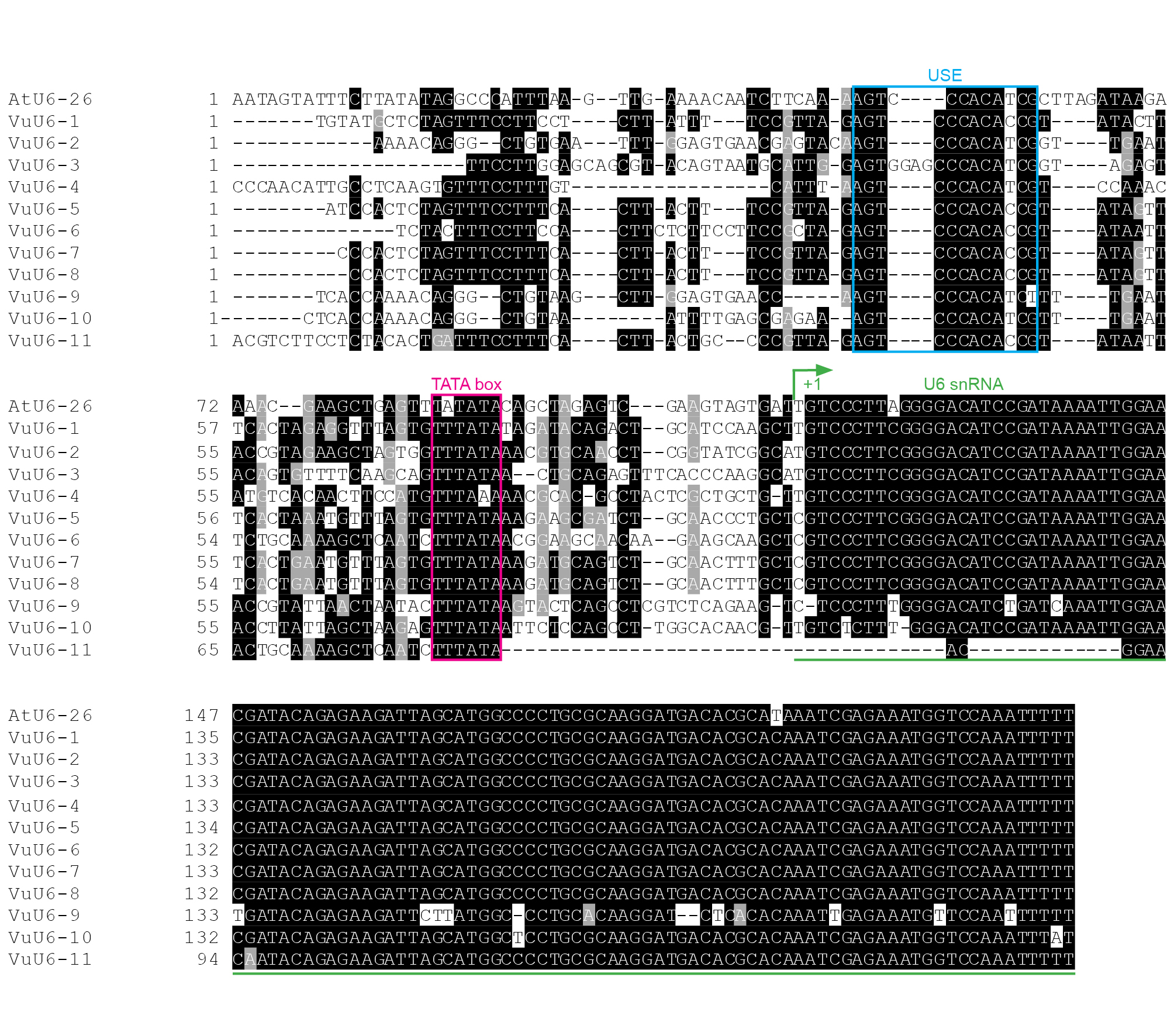

Supplement: Supplementary file 5 — Additional file 5: Fig. S5. Alignment of 11 cowpea U6 and Arabidopsis U6-26 sequences. Conserved elements common to plant U6 promoters; upstream sequence element (USE), TATA-box and U6 small nuclear RNA (snRNA) sequence regions are highlighted in cyan, magenta and green respectively. [file 13007_2020_630_MOESM5_ESM.jpg]
