# Supplementary material for: A detached leaf assay for testing transient gene expression and gene editing in cowpea (Vigna unguiculata [L.] Walp.)
Source: Plant Methods. 2020 Jun 15;16:88. doi: 10.1186/s13007-020-00630-4 (PMC7296760; doi:10.1186/s13007-020-00630-4)
Supplement: Supplementary file 3 — Additional file 3: Fig. S3. Splice variants of the OSD1, REC8 and SPO11-1 genes identified in ovules at different developmental stages in cowpea. Refer to Additional File 6: Table S5 for more details. a-c Exons are shown as grey rectangles, while introns are represented by black lines. Different splice variants are shown underneath the exon-intron organization of a OSD1, b REC8 and c SPO11-1. d Semi-quantitative RT-PCR analysis of OSD1, REC8 and SPO11-1 mRNA. 1 µl of each cDNA was used for the PCR reaction. In the case of SPO11-1, a smear is visible representing a mixture of splicing variants. e Summary of results indicating the number of clones analyzed for each of three genes and the corresponding number of splice forms identified in reproductive tissues. Legend: fTET, female meiotic tetrads; MES, mature embryo sacs; MMC, differentiated megaspore mother cells. [file 13007_2020_630_MOESM3_ESM.pptx]

## Slide 1
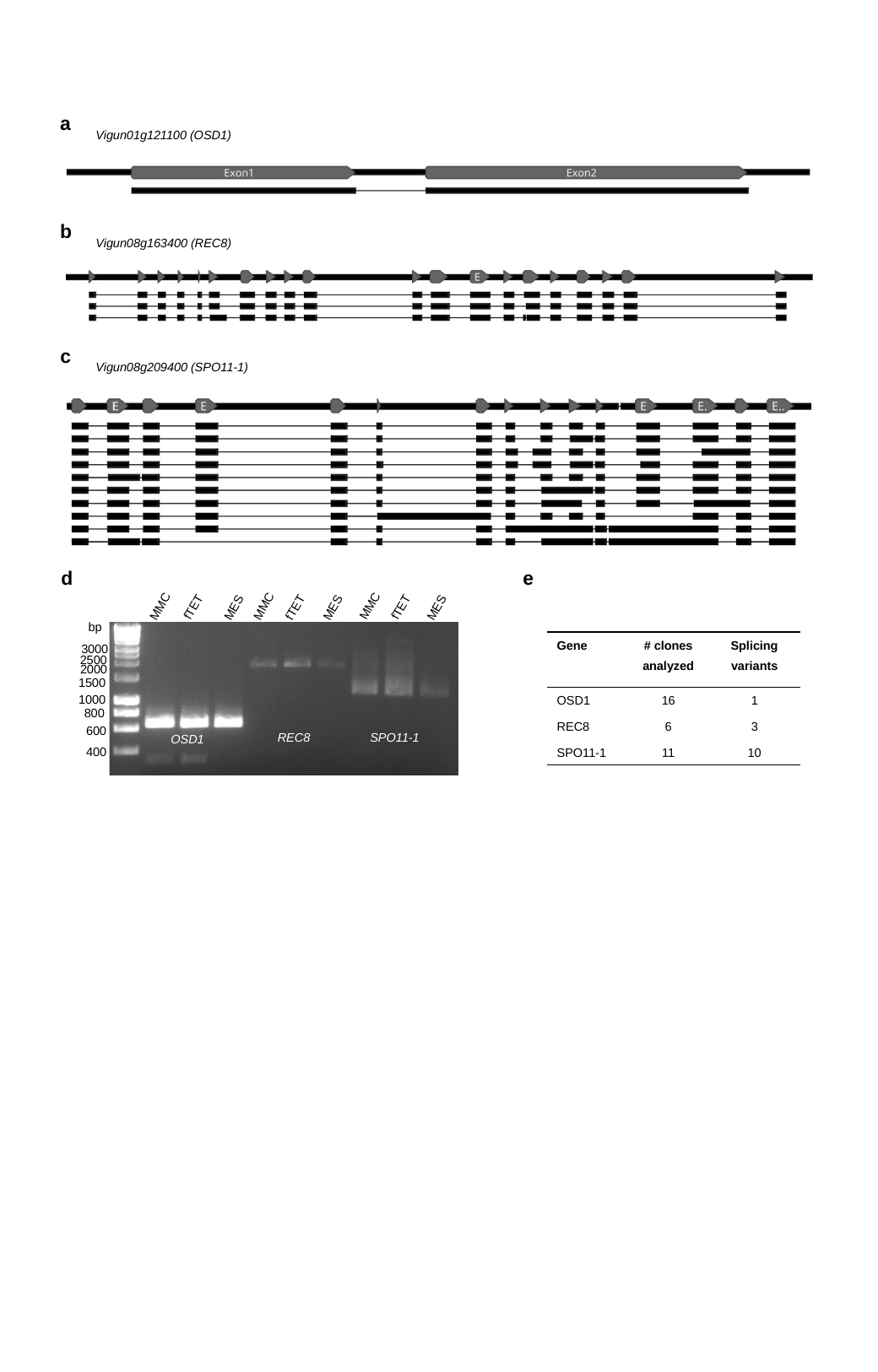

a
Vigun01g121100 (OSD1)
b
Vigun08g163400 (REC8)
c
Vigun08g209400 (SPO11-1)
MES
MMC
fTET
MES
MMC
fTET
MES
fTET
MMC
SPO11-1
REC8
OSD1
bp
3000
2500
2000
1500
1000
800
600
400
d			 e
| Gene | # clones analyzed | Splicing variants |
| --- | --- | --- |
| OSD1 | 16 | 1 |
| REC8 | 6 | 3 |
| SPO11-1 | 11 | 10 |
